# Balancing Graft Survival and Neural Outcomes: Insights From a Systematic Review of Tacrolimus-Induced Neurotoxicity

**DOI:** 10.7759/cureus.94637

**Published:** 2025-10-15

**Authors:** Prithi Dathi, Sonika Gopathoti, Arnav Nandyala, Sahithi Burra, Johnesh Gopathoti, Sai Pavitra Paidimarri

**Affiliations:** 1 Internal Medicine, Siddhartha Medical College, Vijayawada, IND; 2 Internal Medicine, ESIC Medical College and Hospital, Hyderabad, IND; 3 Internal Medicine, Osmania Medical College, Hyderabad, IND; 4 Internal Medicine, Gandhi Medical College, Secunderabad, IND; 5 Internal Medicine, Tianjin Medical University, Tianjin, CHN

**Keywords:** calcineurin inhibitors, extended release dosing, immunosuppression, neurotoxicity, pharmacology, psychosis, tacrolimus, transplantation, tremor, visual evoked potential

## Abstract

Tacrolimus, the most widely used immunosuppressant derived from the fungus *Streptomyces tsukubaensis*, is essential in preventing rejection in solid organ transplants. It is reported to have neurotoxic effects in about 30% of the patients, which include tremors, headache, seizures, psychosis, loss of vision, posterior reversible encephalopathy syndrome (PRES), and coma. This systematic review, adhering to the Preferred Reporting Items for Systematic Reviews and Meta-Analyses (PRISMA) 2020 guidelines, analyzed 13 high-quality studies between 2015 and 2025 identified from PubMed, ScienceDirect, MDPI, and Google Scholar, including observational studies and excluding case reports and articles not written in English. Pathogenesis involved in neurotoxicity is not fully understood but includes the disruption of the blood-brain barrier and the predisposing factors such as drug exposure, twice-daily dosing, CYP3A5 polymorphisms, older age, female sex, Black ethnicity, high model for end-stage liver disease (MELD) scores, pretransplant hemoglobin, and inflammatory markers such as C-reactive protein* *(CRP). Adverse effects are more frequently seen in the early post-transplant period, even at therapeutic levels, and are often associated with dose formulations and dose intervals. CYP3A5 polymorphisms for drug dosing and visual evoked potentials for optic neuropathy can be used as early subclinical markers. Management involves dose reduction or switching to cyclosporine, resulting in resolution of the symptoms. Future multi-center studies are needed to develop standardized diagnostics and personalized strategies to balance immunosuppression and neurological safety.

## Introduction and background

Tacrolimus, a calcineurin inhibitor produced by the soil fungus *Streptomyces tsukubaensis*, is a potent immunosuppressive agent used in the prevention of graft rejection post-transplantation [1]. Despite its broad spectrum of systemic adverse effects, tacrolimus remains the first-line drug administered in solid organ transplant recipients. It has been found that nearly 30% of the patients undergoing tacrolimus therapy experience neurological symptoms ranging from mild and self-limiting to severe and life-threatening [2,3]. Some of the reported neurological effects include tremors, ataxia, headaches, visual disturbances [4], focal neurological deficits, cortical blindness, altered mental status, encephalopathy (including posterior reversible encephalopathy syndrome (PRES)) [5,6], seizures [7], and coma, among others.

The neurotoxic effects of tacrolimus appear to arise from several overlapping mechanisms rather than a single pathway. One proposed explanation is an inflammatory process in which the drug disrupts the blood-brain barrier, injures oligodendrocytes, and leads to demyelination and cerebral edema [8,9]. Another possibility is a vascular mechanism, where tacrolimus alters the balance between prostacyclin and thromboxane, promoting vasoconstriction and ischemia [9]. Individual susceptibility also seems important; for example, variations in the ABCB1 gene that encodes the P-glycoprotein efflux pump may impair drug clearance from the central nervous system, allowing toxic levels to build up even when blood concentrations are within the therapeutic range. High systemic concentrations of tacrolimus can worsen endothelial dysfunction [10] and further reduce the activity of efflux transporters at the blood-brain barrier, enhancing drug entry into the brain. Beyond these processes, tacrolimus is thought to interfere directly with calcium-dependent signaling in neurons and glial cells [11], which may represent a central mechanism of its neurotoxic profile.

Although tacrolimus-induced neurotoxicity (TIN) has been reported across various transplant populations and clinical contexts, there remains a lack of consensus regarding its true incidence, risk factors, and optimal management strategies. Current data are scattered across case reports, observational studies, and small clinical series, highlighting the need for a systematic synthesis of the evidence.

This review aims to comprehensively evaluate the available literature on TIN, with a focus on its clinical presentation, underlying mechanisms, diagnostic approaches, and therapeutic considerations. By consolidating current knowledge, this work seeks to enhance awareness, support early recognition, and inform clinical decision-making in patients receiving tacrolimus therapy.

## Review

Methods

Our review methodology adhered to the principles outlined in the Preferred Reporting Items for Systematic Reviews and Meta-Analyses (PRISMA) 2020 statement for systematic reviews [12].

Search Sources and Strategy

We searched PubMed, Science Direct, MDPI, and Google Scholar to identify relevant studies. Different combinations of keywords and Boolean operators, including "Tacrolimus" and "Neurotoxicity", were applied across these databases. For PubMed, a MeSH-based strategy was developed using the terms "tacrolimus/adverse effects" AND "tacrolimus/neurotoxicity syndromes". The number of articles retrieved from each source is summarized in Table 1.

**Table 1:** Search Strategy

Search Strategy/Keywords	Database	Number of Articles
("Tacrolimus/adverse effects"(Majr)). AND. ("Tacrolimus/Neurotoxicity Syndromes"(Majr))	MeSH-PubMed	5
Tacrolimus. AND. Neurotoxicity	PubMed	65
((Tacrolimus[Text Word]) AND (Neurotoxicity[Title/Abstract])) AND (("2015/01/01"[Date - Publication] : "2025"[Date - Publication]))	PubMed	124
Tacrolimus AND Neurotoxicity	ScienceDirect	300
Tacrolimus AND Neurotoxicity	MDPI	3
Tacrolimus AND Neurotoxicity	Google Scholar	5009

Selection Process

All retrieved articles were imported into EndNote, and duplicate records were removed. Titles and abstracts were screened for relevance, and full texts were reviewed according to the predefined inclusion and exclusion criteria. Studies that satisfied these criteria were included in the final analysis.

Inclusion and Exclusion Criteria

We included studies published between 2015 and 2025 that examined tacrolimus-related neurotoxicity in both adult and pediatric patients. Eligible articles were restricted to human research and comprised randomized controlled trials, observational studies, and systematic and narrative reviews, with full-text access available in English. Studies were excluded if they involved animals and were case reports, gray literature, non-English publications, or released prior to 2015. Articles with pagination restrictions were also not considered.

Quality Assessment

We applied appropriate critical appraisal tools to assess study quality. Randomized controlled trials were reviewed using the Cochrane risk of bias tool [13], while observational studies were examined with the Newcastle-Ottawa scale (NOS) [14]. Systematic reviews were evaluated with AMSTAR [15], and narrative reviews were assessed using SANRA [16]. Only studies that achieved a quality score greater than 70% were included in the final analysis. A detailed summary of the included studies and the appraisal methods used is presented in Tables 2-6.

**Table 2:** NOS for Cross-Sectional Studies NOS - Newcastle-Ottawa Scale

Criteria	Campagne et al. [17]	Riemersma et al. [18]
Selection	-	-
Representativeness	1	1
Sample size	1	1
Ascertainment of exposure	1	1
Non-respondants	0	0
Comparability	-	-
Control of confounding	2	2
Outcome	-	-
Assessment of outcome	1	1
Statistical analysis	1	1
Data completeness	1	1
Total	8/9	8/9

**Table 3:** NOS for Cohort Studies NOS - Newcastle-Ottawa Scale

Criteria	Duman et al. [19]	Lué et al. [20]	Sirek et al. [21]	Alissa et al. [22]
Selection	-	-	-	-
Representativeness of exposed cohort	1	1	1	1
Representativeness of non-exposed cohort	1	1	1	1
Ascertainment of exposure	1	1	1	1
Demonstration that the outcome of interest was not present at the start of the study	1	1	1	1
Comparability	-	-	-	-
Comparability of cohorts on the basis of the design or analysis	1	1	1	1
Comparability of cohort in terms of confounding factors	1	1	1	0
Outcome	-	-	-	-
Assessment of outcome	0	1	1	1
Follow up long enough for outcomes to occur	1	1	1	1
Adequacy of follow-up cohorts	1	1	1	1
Total	8/9	9/9	9/9	8/9

**Table 4:** SANRA Tool for Narrative Reviews SANRA - Scale for the Assessment of Narrative Review Articles

Study	Justification of the article's importance for the readership	Statement of concrete aims or formulation of questions	Description of the literature search	Referencing	Scientific reasoning	Appropriate presentation of data	Total
Hill et al. [23]	2	1	2	2	1	2	10/12
Molema et al. [24]	2	2	1	2	2	2	11/12
Ong et al. [25]	2	1	0	2	2	2	9/12
Kaye et al. [26]	2	1	0	2	2	2	9/12

**Table 5:** The Cochrane Bias Tool for Randomized Controlled Trials (RCTs)

Criteria	Mahaparn et al. [27]
Randomization process (selection bias)	Unclear risk
Deviations from intended interventions (performance bias)	Low risk
Missing outcome data (attrition bias)	Low risk
Measurement of the outcome (detection bias)	Low risk
Selection of the reported result (reporting bias)	Low risk

**Table 6:** AMSTAR Tool for Systematic Reviews AMSTAR - A MeaSurement Tool to Assess systematic Reviews

AMSTAR Criteria	Belur et al. [28]	King et al. [29]
A priori design	Yes	yes
Duplicate study selection and data extraction	Yes	yes
Comprehensive literature review	Yes	yes
Unpublished grey reports sought	No	yes
List of included and excluded studies provided	Yes	yes
Characteristics of individual studies provided	Yes	yes
Scientific quality of studies assessed and documented	No	yes
Scientific quality of studies used for conclusion	No	yes
Statistical method appropriate	Partially yes	yes
Likelihood of publication bias assessed	No	no
Conflict of interest declared in both systematic review and included studies	Yes	yes
Total score	7/11	10/11

Data Collection

The final list of articles obtained through manual selection underwent quality analysis to provide the necessary information for the systematic review.

Results

Study Identification and Selection

**Figure 1:**
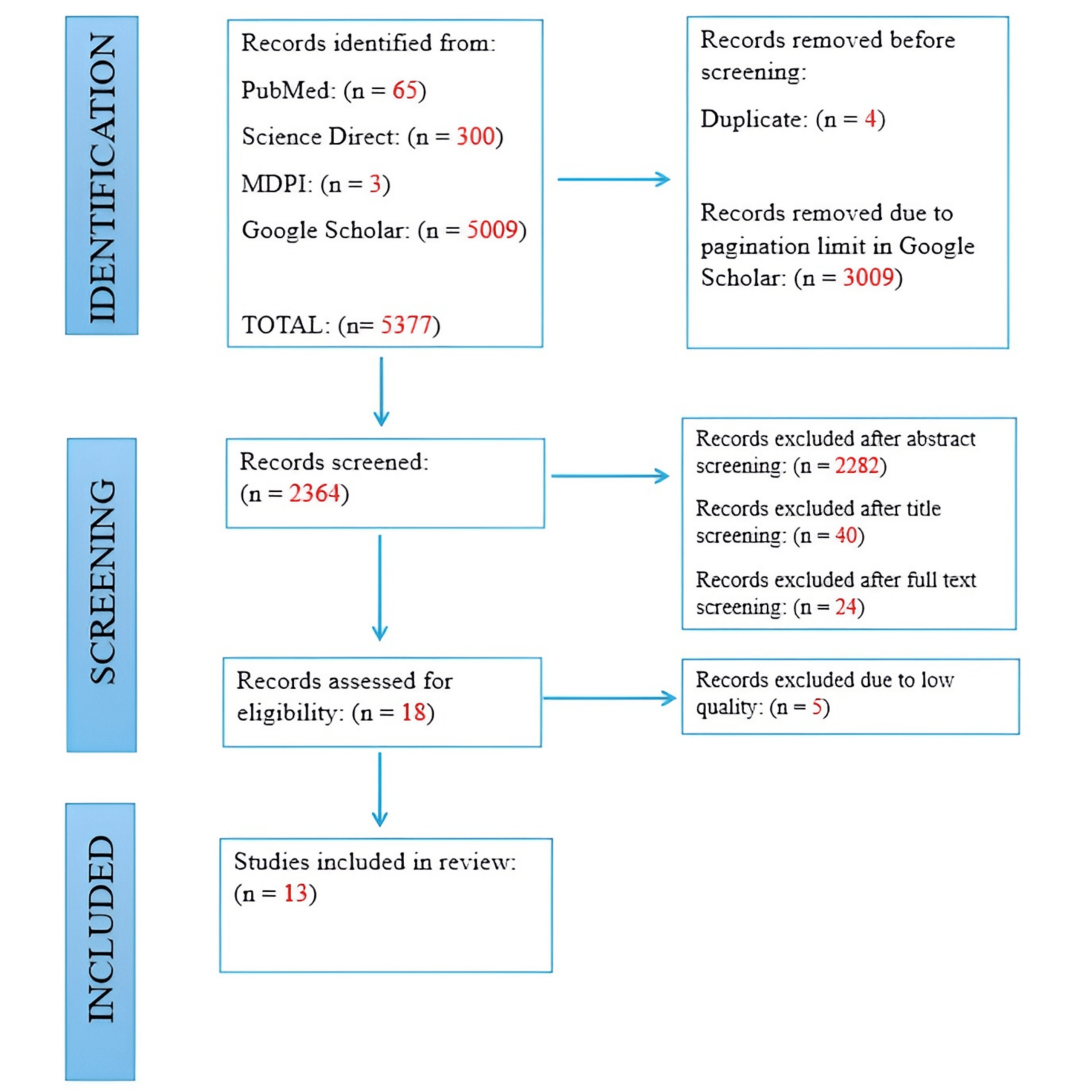
PRISMA Flowchart PRISMA - Preferred Reporting Items for Systematic Reviews and Meta-Analyses

A total of 5,377 records were initially identified across the selected databases and registers. Four duplicate entries were removed using EndNote, and 3,009 records from Google Scholar were excluded due to pagination restrictions. The remaining studies were screened by title and abstract, leading to the exclusion of 2,322 articles that were not relevant to the research topic. This process narrowed the pool to 42 articles, of which 24 were excluded following full-text review. Eighteen studies were then assessed for methodological quality, and 13 were ultimately included in the systematic review. The review process adhered to the PRISMA checklist to maintain transparency and reliability, with the overall workflow summarized in Figure 1.

Study Characteristics

We reviewed the final 13 articles, two cross-sectional studies [17,18], four cohort studies [19-22], four narrative reviews [23-26], one randomized controlled trial [27], and two systematic reviews [28,29]. Table 7 summarises all 13 papers and their characteristics.

**Table 7:** Characteristics of the Studies AUC - Area Under Curve; GI - Gastrointestinal; AEs - Adverse Effects; ADL - Activities of Daily Living; SOTR - Solid-Organ Transplant Recipient; CIIN - Calcineurin Inhibitor Nephrotoxicity; VEP - Visual Evoked Potential; TIN - Tubulointerstitial Nephritis; ECIIN - Early Calcineurin Inhibitor-Induced Neurotoxicity; CNI - Calcineurin Inhibitor; PRES - Posterior Reversible Encephalopathy Syndrome; IR -Immediate Release; LCP - Long-Acting, Controlled Release; CBF - Cerebral Blood Flow

Author and Year of Publication	Type of Study	Quality Appraisal Tool Used	Number of Total Patients/Participants	Aim of the Study	Summary and Interpretation
Campagne et al. 2019 [17]	Cross-Sectional Study	Newcastle-Ottawa Scale (NOS)	67 stable adult renal transplant recipients (African American and Caucasian)	To evaluate the association between tacrolimus exposure (clearance and dose-normalized AUC) and standardized extrarenal adverse events in renal transplant recipients	Approximately 76% of recipients reported gastrointestinal or neurological adverse effects, with frequencies and severities ranging from low to high.
Riemersma et al. 2023 [18]	Cross-Sectional Study	Newcastle-Ottawa Scale (NOS)	689 solid organ transplant recipients (SOTR)	To determine the prevalence and intensity of tremor-related impairment in daily activities among solid organ transplant recipients (SOTR)	Tremor, whether mild or severe, was commonly associated with impaired daily functioning in solid organ transplant recipients, with tacrolimus trough levels identified as a major contributing factor.
Duman et al. 2019 [19]	Cohort Study	Newcastle-Ottawa Scale (NOS)	144 liver transplant recipients	To assess the relationship between tacrolimus blood levels and associated neurotoxicity	Lower tacrolimus blood level z-scores were associated with possible tacrolimus-induced neurotoxicity.
Lué et al. 2019 [20]	Retrospective Cohort Study	Newcastle-Ottawa Scale (NOS)	175 liver transplants in 162 patients	To assess the incidence, risk factors, and clinical outcomes of calcineurin inhibitor-induced neurotoxicity post liver transplantation	CIIN after liver transplantation was common (especially with increased donor age and high pre-transplant sodium) and required more extended hospitalization.
Sirek et al. 2022 [21]	Prospective Cohort Study	Newcastle-Ottawa Scale (NOS)	35 kidney transplant recipients	To evaluate the use of visual evoked potentials in monitoring tacrolimus-induced neurotoxicity post-kidney transplantation	Worsening optic nerve function was indicated with long-term tacrolimus use. VEP was found suitable for the early detection of TIN
Alissa et al. 2022 [22]	Retrospective Cohort Study	Newcastle-Ottawa scale	338 adult liver transplant patients	To identify the incidence and risk factors of tacrolimus-induced neurotoxicity in Saudi patients post liver transplant	Among 338 liver transplant patients, 19% developed ECIIN, predominantly with seizures, agitation, psychosis, and tremors, often requiring a switch to cyclosporine or dose reduction.
Hill et al. 2025 [23]	Case Report and Narrative Review	Scale for the Assessment of Narrative Reviews (SANRA)	-	To present a case of tacrolimus-related pontine neurotoxicity in a child and review similar paediatric cases	This review indicated that most pediatric cases improved after discontinuing tacrolimus and switching to alternative immunosuppression.
Molema et al. 2020 [24]	Case Reports and Narrative Review	Scale for Assessment of Narrative Reviews (SANRA)	32 patients (2 patients from Erasmus MC and 30 patients evaluable with CNI use from the literature)	To investigate the occurrence of CNI-induced neurotoxicity in methylmalonic acidemia patients post hepatic and renal transplantation	22% developed definite CNI toxicity (9% PRES). Early recognition and CNI dose reduction discontinuation are essential to prevent neurological damage.
Ong et al. 2021 [25]	Narrative Review	Scale for the Assessment of Narrative Reviews (SANRA)	-	To review the role of tacrolimus in organ transplantation	This review highlights the superior efficacy and tolerability of tacrolimus in post-transplantation use, along with risks such as nephrotoxicity and neurotoxicity
Kaye et al. 2024 [26]	Narrative Review	Scale for the Assessment of Narrative Reviews (SANRA)	-	To review tacrolimus and mycophenolate-associated toxicities in post-transplant patients	Tacrolimus use was found to be commonly associated with nephrotoxicity, neurotoxicity, cardiotoxicity, and metabolic complications.
Mahaparn et al. 2023 [27]	Randomized Controlled Trial (RCT)	Cochrane Risk of Bias	30 kidney transplant patients	To assess whether IR to LCP conversion of tacrolimus affects CBF, cerebrovascular kinetics, or cognitive function	Switching from IR to LCP tacrolimus was found to improve cerebral blood flow, cerebrovascular regulation, and cognitive function in kidney transplant recipients.
Belur et al. 2025 [28]	Systematic Review	Assessment of Multiple Systematic Reviews (AMSTAR)	-	To study the clinical presentation, risk factors, prognosis, and management of tacrolimus-induced psychosis in liver transplant recipients	This study highlights the need for a standardised definition and management guidelines for tacrolimus-induced psychosis
King et al. 2024 [29]	Systematic Review	Assessment of Multiple Systematic Reviews (AMSTAR)	4030 patients involved in 18 studies	To assess the correlation between tacrolimus exposure and new-onset tremor, headache, and insomnia experienced by adult kidney transplant recipients	This review emphasizes the importance of standardized tremor assessment and the need for further research into its multifactorial etiology, including immunosuppression and patient-specific factors.

Discussion

Knowledge about TIN was relatively scarce for three decades. Some of the earliest and most consistent symptoms reported with TIN were headaches, seizures, and altered mental status [22]. Through the use of CT, MRI, and other modalities, characteristic manifestations such as psychosis, focal symptoms, coma, and leukoencephalopathy were described [18,28]. However, the incidence rates of the various reported neurological symptoms varied among studies, ranging from 8% to 70% [22]. This can be attributed mainly to study heterogeneity and the multifactorial nature of the calcineurin inhibitor-induced neurotoxicity.

Multifactorial Nature of TIN

TIN remains a frequent and multifactorial complication following liver transplantation, with reported incidences ranging from 19% to 21%. Early manifestations typically occur within the first two weeks and include seizures, agitation, psychosis, confusion, tremors, and coma [22,29]. Identified risk factors include pre-transplant hepatic encephalopathy, higher MELD scores, autoimmune hepatitis, perioperative transfusion with fresh frozen plasma, post-transplant hyponatremia, sepsis, and concomitant neurotoxic medications. At the same time, donor-related factors, such as advanced donor age and higher pre-transplant serum sodium levels, have also emerged as independent predictors [19,20]. Importantly, neurotoxicity may develop even at therapeutic tacrolimus concentrations, underscoring the influence of host and graft-related factors beyond drug levels [19]. Correlation analyses have further suggested that pre-transplant hemoglobin and post-transplant CRP levels are significantly associated with tacrolimus blood levels, highlighting the potential contribution of systemic inflammation and baseline hematologic status to CNS vulnerability [19]. Although tacrolimus neurotoxicity does not appear to increase short-term mortality, it is consistently associated with prolonged ICU and hospital stays and frequently necessitates modification or switching of immunosuppressive regimens [20]. These findings emphasize the need for careful risk stratification and early intervention strategies, particularly in recipients with prior encephalopathy or elderly donor grafts.

Dose-Response Relationship of Tacrolimus and Neurotoxicity

Pharmacokinetic analyses indicate that tacrolimus neurotoxicity is driven more by overall exposure than by trough levels alone. A logistic regression based on exposure-response modeling was applied to evaluate the influence of dose-normalized tacrolimus parameters (AUCss0-12h, Cmax, and CL/F) on various extrarenal adverse events, including diarrhea, dyspepsia, insomnia, acne, skin changes, and the composite neurological adverse event ratio [17]. Importantly, most side effects occur within the first few weeks of therapy, when patients are particularly vulnerable to high fluctuations in exposure. Adverse effects have also been reported more frequently with twice daily (BD) dosing compared to once daily (OD) formulations, likely due to greater peak-trough variability. In contrast, OD dosing appears less neurotoxic [21]. Over 80% of Black kidney transplant recipients are extensive or intermediate tacrolimus (Tac) metabolizers due to the CYP3A5*1 allele, compared with only ~9% of White recipients [26]. As a result, Black patients often require higher daily doses to maintain therapeutic trough levels (C0). This leads to greater Tac exposure (higher C0, Cmax, AUC), which may predispose to neurotoxic effects. Notably, extended-release tacrolimus (LCP) achieves lower peak concentrations with reduced variability, which has been associated with fewer neurotoxic side effects, as well as improved cerebral blood flow and cognitive outcomes compared with immediate-release formulations [27]. Moreover, neurotoxicity may develop even at therapeutic tacrolimus concentrations, underscoring the influence of host- and graft-related factors beyond drug levels and suggesting an idiosyncratic susceptibility [19].To optimize therapeutic monitoring, a Bayesian estimator was developed using population pharmacokinetic models in conjunction with limited sampling strategies, enabling accurate prediction of tacrolimus exposure and supporting AUC-guided individualized dosing [17]. Management in clinical practice typically involves dose reduction or conversion to alternative immunosuppression, most often cyclosporine, which is frequently associated with symptom resolution [23].

Most Common Adverse Effects

Tremor is among the most commonly observed adverse effects of tacrolimus. It usually emerges shortly after the start of maintenance therapy and has been reported in as many as 50% of solid organ transplant recipients receiving the drug [18]. Older age is associated with a higher incidence of tremor [29]. Neurologic adverse effects (tremor, headache, insomnia) occur more frequently in females, with Black females particularly at higher risk compared to other groups [29]. Tacrolimus trough concentration was a primary determinant of tremor, reported to be independently associated with tremor-related impairment among solid organ transplant recipients, confirming a dose-dependent relationship [18]. Extended-release formulations of tacrolimus have demonstrated reduced peak-to-trough fluctuations, a lower incidence of tremor, and better quality of life, emphasizing the importance of formulation selection in limiting neurotoxic effects [27].

Tacrolimus-Induced Psychosis

Tacrolimus-induced psychosis is an uncommon but clinically significant manifestation of neurotoxicity, most frequently arising within the first one to six months after transplantation, with many cases presenting in the initial two to four weeks [28]. The clinical picture is highly variable, encompassing delusions, hallucinations, paranoia, agitation, mania, catatonia, and encephalopathy, often in conjunction with neurological signs such as tremors, seizures, or speech disturbances. Psychosis has been reported both in patients with and without prior psychiatric history, although preoperative hepatic encephalopathy and existing mental health disorders may increase vulnerability [28]. Notably, many cases occurred despite tacrolimus serum levels being within the therapeutic range due to the possible influence of genetic polymorphisms, such as ABCB1 transporter variants, suggesting an idiosyncratic susceptibility [28]. Management typically involves dose reduction or switching to alternative immunosuppression, most often cyclosporine, with adjunctive antipsychotics or benzodiazepines used as needed [23]. Symptoms usually resolve within two to four weeks following intervention, supporting the importance of early recognition and treatment. A key limitation across the literature is the absence of standardized diagnostic criteria for tacrolimus-induced psychosis, underscoring the need for consensus definitions [28]. Clinically, these findings highlight the importance of vigilance during the early post-transplant period, particularly in high-risk patients, and highlight the delicate balance required between controlling neuropsychiatric symptoms and maintaining adequate immunosuppression.

VEP: An Early Subclinical Marker of Tacrolimus-Induced Optic Neuropathy

Visual evoked potentials (VEP) represent a sensitive approach for detecting subclinical TIN. Worsening of P100 latency and amplitude with prolonged therapy, together with correlations between tacrolimus trough levels and P2 wave parameters observed only after long-term exposure, suggest that neurotoxicity is essentially cumulative and driven by chronic rather than acute levels [21]. Evidence indicates that twice-daily formulations may confer greater neurotoxic risk, while once-daily dosing appears less harmful [21]. Importantly, VEP abnormalities can occur in the absence of overt clinical or ophthalmologic findings, highlighting their value as early diagnostic markers [21]. Potential mechanisms include disruption of calcineurin-mediated neuronal processes, blood-brain barrier dysfunction facilitating drug accumulation, and altered brain-derived neurotrophic factor (BDNF) signaling [29]. Collectively, these findings underscore that tacrolimus can induce neurotoxic effects even within therapeutic ranges and support the use of VEP monitoring to guide dose adjustments or conversion to less neurotoxic agents [19].

Limitations

The main limitation of this review is the limited availability of high-quality studies on TIN, which reduces the strength of evidence that can be drawn. While randomized controlled trials were included, case reports and case series (often valuable for capturing rare or early presentation) were excluded, which may have limited the clinical scope of the findings. Furthermore, although 5,009 articles were initially identified through Google Scholar, only 2,000 could be accessed due to pagination limits, raising the possibility that relevant studies were missed. Finally, this review was restricted to English-language, freely accessible publications, which may further narrow the scope of included evidence.

## Conclusions

This review focuses on how tacrolimus therapy contributes to the development of neurotoxicity in transplant recipients. The studies we reviewed revealed that neurotoxic effects may occur even at therapeutic drug levels, driven by mechanisms such as inhibition of calcineurin-dependent neuronal processes, endothelial injury, and increased penetration across the blood-brain barrier. Patient-specific factors, including age, sex, ethnicity, and genetic polymorphisms, along with drug-related variables such as dose, formulation, and frequency, further influence risk. Common clinical manifestations range from tremor and headache to severe complications, such as seizures, psychosis, and posterior reversible encephalopathy syndrome.

Extended-release and once-daily formulations appear to reduce neurotoxic risk by minimizing fluctuations in exposure, while VEPs have emerged as a sensitive tool for detecting subclinical toxicity. Understanding these mechanisms and risk factors is essential, as they highlight opportunities for early recognition, personalized dosing, and optimized monitoring strategies. This review provides insight into current knowledge on TIN and serves as a resource for future studies aimed at improving the management of this complication. Future multicenter, prospective investigations are warranted to establish standardized diagnostic criteria and to optimize individualized therapeutic strategies that preserve graft survival while minimizing neurological sequelae.

## References

[1] Hussein IM, Micieli JA (2022). Tacrolimus optic neuropathy mimicking papilledema. Case Rep Ophthalmol.

[2] Gmitterová K, Minár M, Žigrai M, Košutzká Z, Kušnírová A, Valkovič P (2018). Tacrolimus-induced parkinsonism in a patient after liver transplantation - case report. BMC Neurol.

[3] Etta PK, Bavikar P, Phani R (2023). Tacrolimus-associated cerebral vasculopathy presenting with recurrent intracerebral hemorrhage. Indian J Nephrol.

[4] Canovai E, Cassiman C, Ceulemans LJ (2020). Tacrolimus-induced optic neuropathy after multivisceral transplantation. Transplant Direct.

[5] Çakmak F, Kanbakan A, Akdeniz YS, İpekçi A, İkizceli T (2019). Tacrolimus-induced vision loss in a renal transplant patient: posterior reversible encephalopathy syndrome. Exp Clin Transplant.

[6] Rivillas JA, Galindo-Coral S, Arias-Mora F, Lopez-Ponce de Leon JD, Florez-Alarcón NA, Olaya-Rojas P, Gomez-Mesa JE (2021). Posterior reversible encephalopathy syndrome associated with tacrolimus in cardiac transplantation. Case Rep Cardiol.

[7] Liu JF, Shen T, Zhang YT (2020). Posterior reversible encephalopathy syndrome and heart failure tacrolimus-induced after liver transplantation: a case report. World J Clin Cases.

[8] Vangala S, Beebani G, Thiem R, Dereczyk A (2020). Mania associated with supratherapeutic tacrolimus levels in a patient with no psychiatric history. Psychosomatics.

[9] Rasool N, Boudreault K, Lessell S, Prasad S, Cestari DM (2018). Tacrolimus optic neuropathy. J Neuroophthalmol.

[10] Kapoor A, Birks E, Lenneman A, McCants K (2017). Posterior reversible encephalopathy syndrome after heart transplantation: diagnosis and immunosuppressive therapy. Tex Heart Inst J.

[11] Gomes GF, Batista CR, Silva MC (2025). Tacrolimus mitigates pathological patterns in mouse models of Alzheimer's disease. Biomed Pharmacother.

[12] Page MJ, McKenzie JE, Bossuyt PM (2021). The PRISMA 2020 statement: an updated guideline for reporting systematic reviews. BMJ.

[13] Flemyng E, Moore TH, Boutron I, Higgins JP, Hróbjartsson A, Nejstgaard CH, Dwan K (2023). Using risk of bias 2 to assess results from randomised controlled trials: guidance from Cochrane. BMJ Evid Based Med.

[14] Wells GA, Shea B, O’Connell D, Peterson J, Welch V, Losos M, Tugwell P (2025). The Newcastle-Ottawa scale (NOS) for assessing the quality of nonrandomised studies in meta-analyses. https://www.ohri.ca/programs/clinical_epidemiology/oxford.asp.

[15] Shea BJ, Grimshaw JM, Wells GA (2007). Development of AMSTAR: a measurement tool to assess the methodological quality of systematic reviews. BMC Med Res Methodol.

[16] Baethge C, Goldbeck-Wood S, Mertens S (2019). SANRA—a scale for the quality assessment of narrative review articles. Res Integr Peer Rev.

[17] Campagne O, Mager DE, Brazeau D, Venuto RC, Tornatore KM (2019). The impact of tacrolimus exposure on extrarenal adverse effects in adult renal transplant recipients. Br J Clin Pharmacol.

[18] Riemersma NL, Kremer D, Knobbe TJ (2023). Tremor, daily functioning, and health-related quality of life in solid organ transplant recipients. Transpl Int.

[19] Duman B, Herdi O, Kirimker EO (2019). The association between tacrolimus blood levels and possible neurotoxicity following liver transplantation. Ann Med Res.

[20] Lué A, Martinez E, Navarro M (2019). Donor age predicts calcineurin inhibitor induced neurotoxicity after liver transplantation. Transplantation.

[21] Sirek S, Kolonko A, Pojda-Wilczek D (2022). Visual evoked potentials as a method for the prospective assessment of tacrolimus neurotoxicity in patients after kidney transplantation. Doc Ophthalmol.

[22] Alissa DA, Alkortas D, Alsebayel M (2022). Tacrolimus-induced neurotoxicity in early post-liver transplant Saudi patients: incidence and risk factors. Ann Transplant.

[23] Hill A, Haradwala MB, Le Pichon JB (2025). Tacrolimus-related neurotoxicity of the pons in children: review of the literature and a case report. Ann Child Neurol Soc.

[24] Molema F, Williams M, Langendonk J (2020). Neurotoxicity including posterior reversible encephalopathy syndrome after initiation of calcineurin inhibitors in transplanted methylmalonic acidemia patients: two case reports and review of the literature. JIMD Rep.

[25] Ong SC, Gaston RS (2021). Thirty years of tacrolimus in clinical practice. Transplantation.

[26] Kaye AD, Shah SS, Johnson CD (2024). Tacrolimus- and mycophenolate-mediated toxicity: clinical considerations and options in management of post-transplant patients. Curr Issues Mol Biol.

[27] Mahaparn I, Lepping RJ, Montgomery RN, Mukherjee R, Billinger SA, Brooks WM, Gupta A (2023). The association of tacrolimus formulation on cerebral blood flow and cognitive function. Transplant Direct.

[28] Belur P, Dani K, Cho SH (2025). Tacrolimus-induced psychosis in liver transplant recipients: a systematic review of all published cases. J Liver Transplant.

[29] King CP, Cossart AR, Isbel NM, Campbell SB, Staatz CE (2024). The association between tacrolimus exposure and tremor, headache and insomnia in adult kidney transplant recipients: a systematic review. Transplant Rev (Orlando).

